# Expansion of the mangrove species *Rhizophora mucronata* in the Western Indian Ocean launched contrasting genetic patterns

**DOI:** 10.1038/s41598-021-84304-8

**Published:** 2021-03-02

**Authors:** Ludwig Triest, Tom Van der Stocken, Dennis De Ryck, Marc Kochzius, Sophie Lorent, Magdalene Ngeve, Hajaniaina Andrianavalonarivo Ratsimbazafy, Tim Sierens, Rosa van der Ven, Nico Koedam

**Affiliations:** 1 Ecology and Biodiversity, Vrije Universiteit Brussel, Pleinlaan 2, 1050 Elsene, Brussels, Belgium; 2 Marine Biology, Vrije Universiteit Brussel, Pleinlaan 2, 1050 Elsene, Brussels, Belgium; 3 Department of Plant Sciences and Landscape Architecture, University of Maryland, College Park, MD 20742 USA; 4 Laboratory of Systems Ecology and Resource Management, Département de Biologie Des Organismes, Université Libre de Bruxelles - ULB, Av. F.D. Roosevelt 50, CPi 264/1, 1050 Brussels, Belgium; 5 Marine Animal Ecology Group, Wageningen University, Wageningen, The Netherlands

**Keywords:** Molecular ecology, Plant ecology

## Abstract

Estimates of population structure and gene flow allow exploring the historical and contemporary processes that determine a species’ biogeographic pattern. In mangroves, large-scale genetic studies to estimate gene flow have been conducted predominantly in the Indo-Pacific and Atlantic region. Here we examine the genetic diversity and connectivity of *Rhizophora mucronata* across a > 3,000 km coastal stretch in the Western Indian Ocean (WIO) including WIO islands. Based on 359 trees from 13 populations and using 17 polymorphic microsatellite loci we detected genetic breaks between populations of the (1) East African coastline, (2) Mozambique Channel Area (3) granitic Seychelles, and (4) Aldabra and northern Madagascar. Genetic structure, diversity levels, and patterns of inferred connectivity, aligned with the directionality of major ocean currents, driven by bifurcation of the South Equatorial Current, northward into the East African Coastal Current and southward into the Mozambique Channel Area. A secondary genetic break between nearby populations in the Delagoa Bight coincided with high inbreeding levels and fixed loci. Results illustrate how oceanographic processes can connect and separate mangrove populations regardless of geographic distance.

## Introduction

In coastal and marine systems, where ocean currents may transport individuals over 100–1000 s of kilometres, population genetic approaches strongly promoted connectivity research during the last three decades. Interestingly, a recent quantitative assessment of this research revealed important taxonomic and geographical biases, with mangrove population connectivity having received minimal attention compared to for example fish, seagrasses and corals^1^. Efforts to fill this gap are valuable, as connectivity determines the genetic diversity and structure of populations, increases the effective population size, while high connectivity can replenish degraded sites. Insights in connectivity are therefore essential for effective conservation management. In a broader perspective, a better qualitative and quantitative understanding of mangrove connectivity would provide an interesting biogeographic comparison with systems that have a circumtropical overlap in their range but show distinct differences in their dispersal traits (e.g., development during dispersal, buoyancy behaviour, and viability).

Mangroves consist of woody salt-tolerant plants that are morphologically and eco-physiologically adapted to the dynamic conditions of the intertidal zone. They are found on coastal, estuarine and deltaic tidal flats, predominantly at tropical and subtropical latitudes^2^. The global spatial coverage of mangroves is estimated at ~ 137,000 km^2^, spanning the territories of more than 100 countries^3^. Nevertheless, the consequences of climate change such as accelerated sea-level rise, storm intensification, and changes in temperature and precipitation regimes^4^, as well as human activities, such as urbanization, aquaculture and agriculture^5^, alter the distributional range of mangrove forests.

Gene flow in mangroves is determined by insect, wind, and bird pollination^6,7^ and the dispersal of water-buoyant propagules (essentially ‘seedlings’) via near-shore, coastal, and open-ocean surface currents^8^. While pollination operates at local scales, propagule floating and viability periods of several months^8,9^ allow for local-to-global scale dispersal and connectivity^10–12^. The spatial scale of connectivity ultimately depends on the additive effect of different factors, such as propagule traits, the timing of propagule release, physical barriers, the availability of suitable habitats, and the spatiotemporal characteristics of the dispersal vectors^13^. Oceanographic features such as eddies and regions of convergence may constrain, delay or prevent gene flow between nearby populations, rendering the isolation-by-distance (IBD) model too simplistic to explain observed genetic differentiation^14^. Using nuclear microsatellite markers and a Lagrangian particle-tracking model, a strong genetic discontinuity was found between *Rhizophora racemosa* G. Mey populations at both sides of an oceanic convergence zone in the Gulf of Guinea^15^. Similarly, genetic structure of *Rhizophora mucronata* Lam. populations along the Malay Peninsula was explained by prevailing ocean currents and the species’ dispersal potential, and not by geographical distance^16,17^. Indeed, as more species are being studied, biogeographical “barriers” seem more permeable than previously thought and act as filters rather than impenetrable barriers^18^.

Although the amount of mangrove genetic and phylogeographic data is increasing, most studies have focused on populations of *Avicennia* and *Rhizophora* species in Southeast Asia^19–24^, Central and South America^25–29^, and East Atlantic^15,30,31^, with only limited attention for mangrove populations in the Western Indian Ocean (WIO) in general (but see^32^). Several large-scale mangrove genetic studies have considered samples from the WIO region^7,12,21^, but the majority of locations and the focus in these studies were centered on the Eastern Indian Ocean (EIO) region.

**Figure 1:**
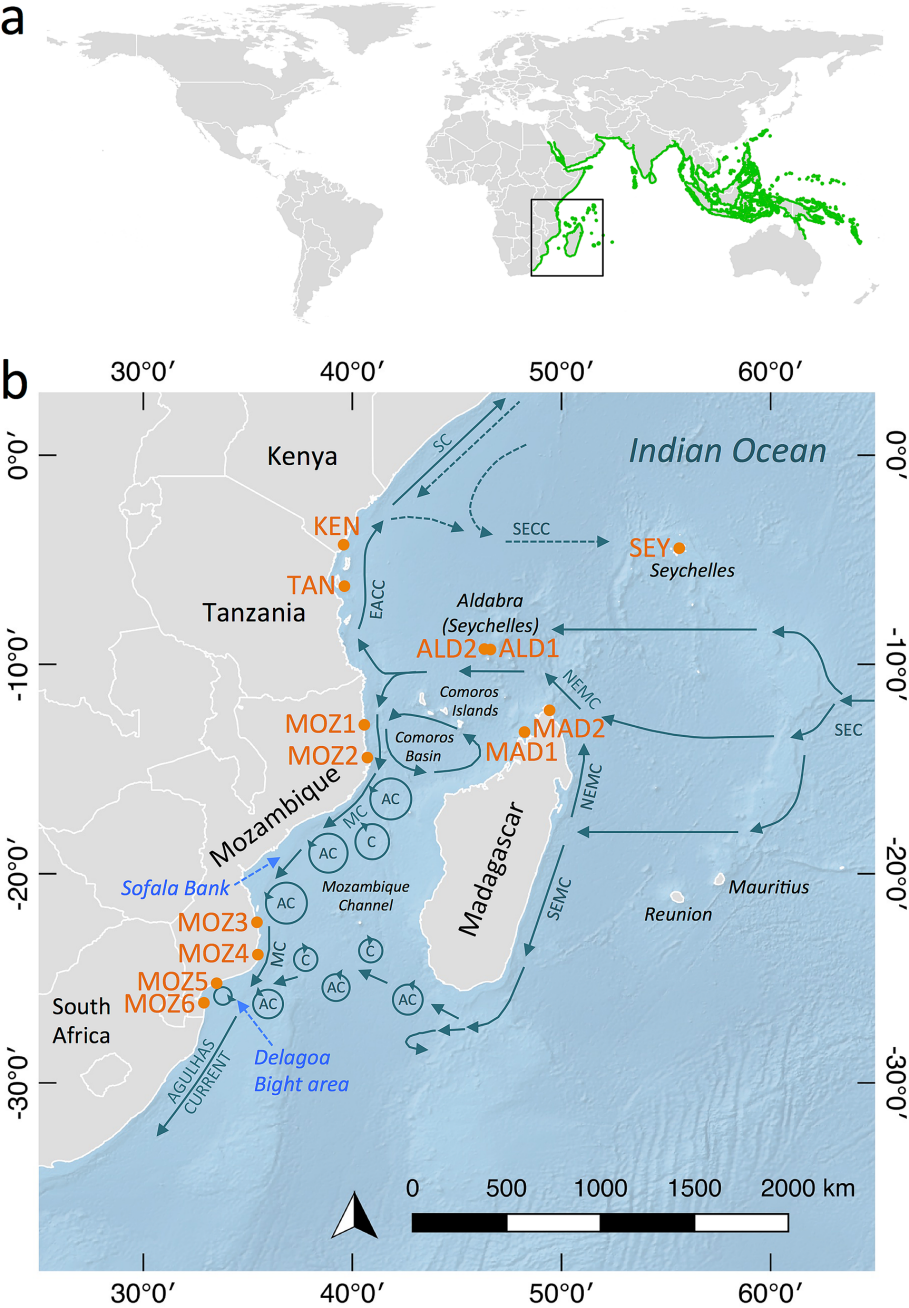
Global distribution of *Rhizophora mucronata* and the dominant ocean circulation in the Western Indian Ocean region where 13 populations were sampled. (**a**) Global distribution of *R.* mucronata^36^ (green) and the study region (black rectangle). (**b**) Schematic representation of the dominant near-surface circulation in the Western Indian Ocean region^51^. North-East and South-East Madagascar Currents (NEMC, SEMC); Mozambique Current (MC), highly irregular in its occurrence^83^; Anti-cyclonic eddies (AC); Cyclonic eddies (C); East Africa Coastal Current (EACC); and the Somali Current (SC) and South Equatorial Current (SEC), during the northeast monsoon (November-February, dotted line) and the southwest (June–September) monsoon season^41^; and the South Equatorial Countercurrent (SECC). Sampled populations of *R. mucronata* are shown in orange. Population codes are denoted in Table 1. The background map was created with the QGIS 3.10.10 software (www.qgis.org), using detailed (1:10 m) ocean bottom relief, land and island polygons, as well as country administrative boundaries provided by Natural Earth (www.naturalearthdata.com).

*Rhizophora mucronata* has a distributional range stretching across mangroves of the Indo-West Pacific (IWP) region, from the East African shorelines to the Western Pacific^33^, with its southern limit in Southeast Africa and its northern limit in the Persian Gulf^34^ (Fig. 1a). The northern limit has been associated with temperature and rainfall tolerances, whereas the range limit in southeast Africa is assumed to be temperature-related and may be influenced also by constraints in dispersal and habitat availability^35^. The natural distribution of *R. mucronata* overlaps with other IWP *Rhizophora* species (*R. apiculata* Blume and *R. stylosa* Griff) in the Western Pacific. It is the only *Rhizophora* species in the WIO^33^ and has one of the widest ranges of all mangrove species. Even-though its global conservation status currently is of ‘least concern’, its global population is decreasing due to residential and commercial development, logging and wood harvesting, agriculture and aquaculture, and potentially climate-induced habitat alteration^36^.

In this study, we aim to quantify the population genetic diversity and structure of *R. mucronata* in the WIO region. This region is characterised by the bifurcation of the South Equatorial Current (SEC) and the complex circulation system of the Mozambique Channel that is dominated by energetic southward moving eddies^37,38^. As a null-hypothesis, we considered that over a broad geographical scale, *R. mucronata* populations in the WIO would comprise a single evolutionarily significant unit and hypothesise that (1) highest genetic diversity levels will be found for populations located in the core region of the species’ distribution, whereas lower levels of genetic diversity will be found for populations on remote small islands; (2) spatial patterns of genetic differentiation will align with patterns of dominant contemporary ocean currents. For this purpose, we sampled 13 *R. mucronata* populations distributed across the western extent of its range (Fig. 1, Table 1) and utilised 17 polymorphic nuclear microsatellite loci. We further discuss how historical events, contemporary ocean surface currents, and elevated local inbreeding levels, may influence estimated patterns of gene flow.

**Table 1:** Location details of 13 *Rhizophora mucronata* populations of the Western Indian Ocean (N** = **sample size).

Code	Country	Location	Latitude	Longitude	N
KEN	Kenya	Gazi Bay	− 4.416749	39.510001	27
TAN	Tanzania	Unguja, Zanzibar	− 6.439767	39.561865	30
MOZ1	Mozambique	Pemba	− 12.977269	40.507873	20
MOZ2	Mozambique	Nacala	− 14.482243	40.653189	34
MOZ3	Mozambique	Vilanculos	− 22.209981	35.394369	41
MOZ4	Mozambique	Inhambane	− 23.795773	35.499341	18
MOZ5	Mozambique	Limpopo	− 25.163395	33.505903	24
MOZ6	Mozambique	Inhaca	− 26.038127	32.902805	30
SEY	Seychelles	Mahé	− 4.613755	55.458582	24
ALD1	Seychelles	La Gigi, Aldabra	− 9.400295	46.210647	30
ALD2	Seychelles	Middle Camp, Aldabra	− 9.373783	46.438728	21
MAD1	Madagascar	Andilana	− 13.252461	48.182015	30
MAD2	Madagascar	Ramena	− 12.254765	49.341469	30

## Results

### Allele and gene diversity

The number of alleles per locus ranged from two to 10 with a total of 84 alleles over all loci and populations. The mean number of alleles reached 2.4 (1.8–3), the effective number of alleles was 1.5 (1.3–2), whereas the allelic richness ranged from 1.7 to 2.8 (at k = 34 gene copies) for comparison of allele diversity between populations (Table 2). East African populations (KEN and TAN) harboured the largest allelic richness. All populations contained several loci with > 2 alleles indicating multiple founders. All observed heterozygosity levels were lower than the expected ones. The u*H*_e_ averaged at 0.271 and ranged per population from 0.148 (MOZ5) to 0.416 (KEN) (Table 2). Significant deviations (p < 0.05) from the Hardy–Weinberg Equilibrium were observed within each of the 13 populations, because of heterozygote deficiency in a varying set of four to 12 loci. The percentage of polymorphic loci per population was 55–100% and the number of fixed loci (either monomorphic or fully homozygous) was high for several southernmost sites in Mozambique (MOZ5 and MOZ6), and remote island locations of Aldabra and Madagascar, though not for the Seychelles (Table 2).

**Table 2:** Genetic diversity measures of 13 *Rhizophora mucronata* populations of the Western Indian Ocean. Highest levels of allele diversity can be found in East Africa (KEN and TAN). Inbreeding is present in all populations with highest levels on the small remote islands (SEY, ALD1 and ALD2). A high number of monomorphic and fixed loci were apparent in southern Africa (MOZ5 and MOZ6) and remote locations (ALD1, ALD2 and MAD1). Estimated selfing rates were generally high. MOZ1 and MOZ2 individuals can be assigned to other populations whereas individuals from SEY were assigned solely to their own population. Total number of alleles (*A*), mean number of alleles (*A*_M_), effective number of alleles (*A*_E_), allelic richness at k = 34 gene copies (*A*_R_), observed heterozygosity (*H*_O_), unbiased expected heterozygosity (u*H*_e_) heterozygosity, inbreeding coefficient (*F*_IS_), number of loci fixed as homozygotes for one or more allele (FixLoc), selfing rate (S), percentage of individuals assigned to own population (%Self) and percentage of individuals assigned to any other population (%Other). Significance levels are indicated as follows: *** significant at p < 0.001, ** significant at p < 0.01, * significant at p < 0.05, and ns: not significant.

Pop	*A*	*A*_M_	*A*_E_	*A*_R_	*H*_O_	u*H*_e_	*F*_IS_	FixLoc	S	%Self	%Other
KEN	48	2.8	2.0	2.7	0.257	0.416	0.387*	2	0.47***	78	22
TAN	52	3.0	1.9	2.8	0.243	0.402	0.401*	0	0.49***	80	20
MOZ1	42	2.5	1.6	2.4	0.193	0.332	0.425*	3	0.02 ns	25	75
MOZ2	49	2.9	1.6	2.6	0.202	0.324	0.381*	0	0.56***	32	68
MOZ3	50	2.9	1.6	2.5	0.155	0.277	0.444*	6	0.56***	66	34
MOZ4	39	2.3	1.5	2.3	0.154	0.264	0.425*	2	0.47*	78	22
MOZ5	30	1.8	1.3	1.7	0.047	0.148	0.690*	9	0.89***	75	25
MOZ6	37	2.2	1.5	2.0	0.127	0.238	0.468*	7	0.71***	80	20
SEY	41	2.4	1.6	2.3	0.140	0.323	0.573*	2	0.75***	100	0
ALD1	39	2.3	1.4	2.1	0.129	0.225	0.431*	6	0.45*	67	33
ALD2	33	1.9	1.4	1.9	0.087	0.186	0.540*	9	0.43 ns	76	24
MAD1	37	2.2	1.3	2.0	0.106	0.183	0.425*	8	0.54**	93	7
MAD2	42	2.5	1.4	2.3	0.143	0.209	0.391*	4	0.63***	73	27
Overall	84	2.4	1.5	2.9	0.152	0.271	0.455***			71	29

The within population inbreeding coefficient *F*_IS_ for the total population was 0.455 (AMOVA, p < 0.001). Within population *F*_IS_ ranged from 0.381 (MOZ2) up to 0.690 (MOZ5) with all populations significantly inbred (Table 2), though with high levels on small remote islands (ALD and SEY). The estimated selfing rates for *R. mucronata* were significantly high in most populations (S = 0.45–0.89), except for MOZ1 and ALD2 (Table 2). Kinship values within populations reached an overall *F*_IJ_ of 0.271.

### Genetic structure

Global inbreeding reached an AMOVA-*F*_IT_ of 0.609 (p < 0.001) and a population differentiation AMOVA-*F*_ST_ of 0.281 (p < 0.001) (Table 3). Global AMOVA-*R*_ST_ was zero, indicating there are no larger differences due to allele size differences, and hence no evolutionary signal among populations within the WIO. A hierarchical AMOVA considering two regions gave the highest regional differentiation (*F*_RT_ = 0.110, p < 0.001) for the grouping of the Aldabra-Madagascar populations (ALD1, ALD2, MAD1, MAD2) versus all other populations. When considering a hierarchy of three regions, highest values (*F*_RT_ = 0.169, p < 0.001) were obtained when grouping East Africa (KEN, TAN), versus Aldabra and Madagascar, versus all other populations (MOZ1-MOZ6, SEY). A hierarchical AMOVA considering four regions (Table 3), namely East Africa (KEN and TAN), the Mozambique Channel (MOZ1 to MOZ6), the Seychelles (SEY), and as a fourth group the Aldabra Atoll and Madagascar, gave the largest variance—among all possible groupings—of WIO populations with *F*_RT_ = 0.185 (p < 0.001). Overall connectivity between populations is very low with an estimated *N*m less than 1. Pairwise *F*_ST_ values between populations varied between 0.002 and 0.529 (for which all values except one case were significant at p < 0.001, Supplementary Table S1). Pairwise allelic differentiation *D*est was congruent with *F*_ST_ (Supplementary Table S1). Strongest differentiation was obtained for Aldabra (ALD1, ALD2) or Madagascar (MAD1, MAD2) against all other sites. Neighbouring sites appear most connected having lowest *F*_ST_ and *D*est (MOZ1 vs. MOZ2; and ALD1 vs. ALD2). A Mantel test for all 13 populations suggested isolation by distance (y = 0.2494x − 0.3533, p = 0.001), but with a low predictability for Euclidean distances (r^2^ = 0.24). A more precise test of IBD was performed at the level of individual pairwise kinship coefficients using four classes of increasing geographic distances. Significantly higher than average kinship values were found for the first distance class up to 260 km—average 136 km—(*F*_IJ_ = 0.164, p < 0.001), and the second distance class up to 500 km—average 385 km—(*F*_IJ_ = 0.088, p < 0.01). The ln-transformed b-slope was − 0.078 (p < 0.001) over a full range of 3008 km.

**Table 3:** Summary of AMOVA and *F*-statistics of *Rhizophora mucronata* for all populations and a hierarchical AMOVA considering four regions, namely East Africa (KEN and TAN), the Mozambique Channel (MOZ1 to MOZ6), the Seychelles (SEY) and as a fourth group the Aldabra Atoll and Madagascar (ALD1, ALD2, MAD1, MAD2). These four regions account for the largest variance of the Western Indian Ocean populations. Overall connectivity between populations is low with an estimated Nm below 1; df: degrees of freedom; SS: sum of squares; MS: mean of squares; % Est.Var.: estimated variance.

No regions	df	SS	MS	Est. Var	%	F-statistics	p-value
Among Populations	12	661.089	55.091	0.938	28	*F*_ST_ = 0.281	0.001
Among individuals	346	1206.579	3.487	1.091	33	*F*_IS_ = 0.455	0.001
Within individuals	359	468.500	1.305	1.305	39	*F*_IT_ = 0.609	0.001
Total	717	2336.169		3.334	100	Nm = 0.6	

Four regions	df	SS	MS	Est. Var	%	F-statistics	p-value
Among regions	3	395.755	131.918	0.651	19	*F*_RT_ = 0.185	0.001
Among populations	9	265.335	29.482	0.472	13	*F*_SR_ = 0.164	0.001
Among individuals	346	1206.579	3.487	1.091	31	*F*_ST_ = 0.319	0.001
Within individuals	359	468.500	1.305	1.305	37	*F*_IS_ = 0.455	0.001
Total	717	2336.169		3.519	100	*F*_IT_ = 0.629	0.001

**Figure 2:**
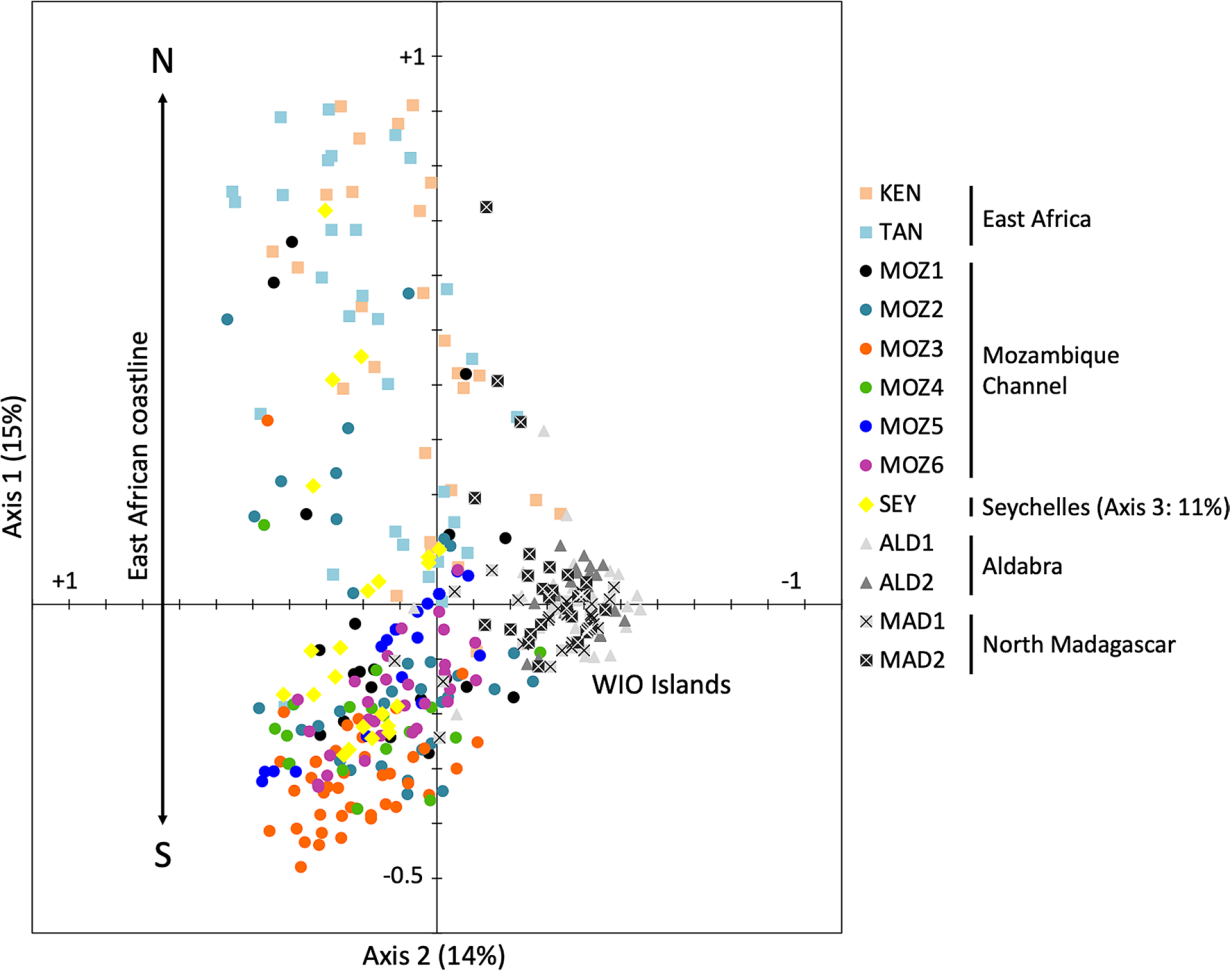
Principal coordinate analysis (PCoA) biplot of individual trees from 13 *Rhizophora mucronata* populations in the Western Indian Ocean region. Both axes explain a nearly similar amount of variance. A first axis reflects the largest differences in allele distribution along the African continent with enhanced diversity in East Africa (KEN and TAN). The variation along the second axis is mainly caused by a high number of fixed loci within individual mangrove trees of remote and island populations (ALD1, ALD2, MAD1 and MAD2). *R. mucronata* trees from the Seychelles (SEY) showed intermediate allele diversity and only few fixed loci and hence did not separate as a group except at the third axis (explaining 11% of the variance). Population codes are denoted in Table 1.

A PCoA of individual genotypic distances had both axes explaining a nearly similar amount of variance (Fig. 2). A first axis (15%) reflected the largest differences in allele distribution along a north-to-south gradient of the East African coast, with enhanced individual tree genotypic diversity in KEN and TAN. Variation along the second axis (14%) is mainly caused by a high number of fixed loci within individual mangrove trees of remote and island populations (ALD1, ALD2, MAD1 and MAD2). *Rhizophora mucronata* trees from the Seychelles showed intermediate allele diversity, only few fixed loci, several nearly private alleles at high frequencies, and hence, did not separate as a group, unless along the third axis (11%). A population assignment test gave an average of 71% of the individuals assigned to their own population. However, MOZ1 and MOZ2 individuals could be assigned largely to other populations, whereas individuals from SEY were assigned solely to their own population.

**Figure 3:**
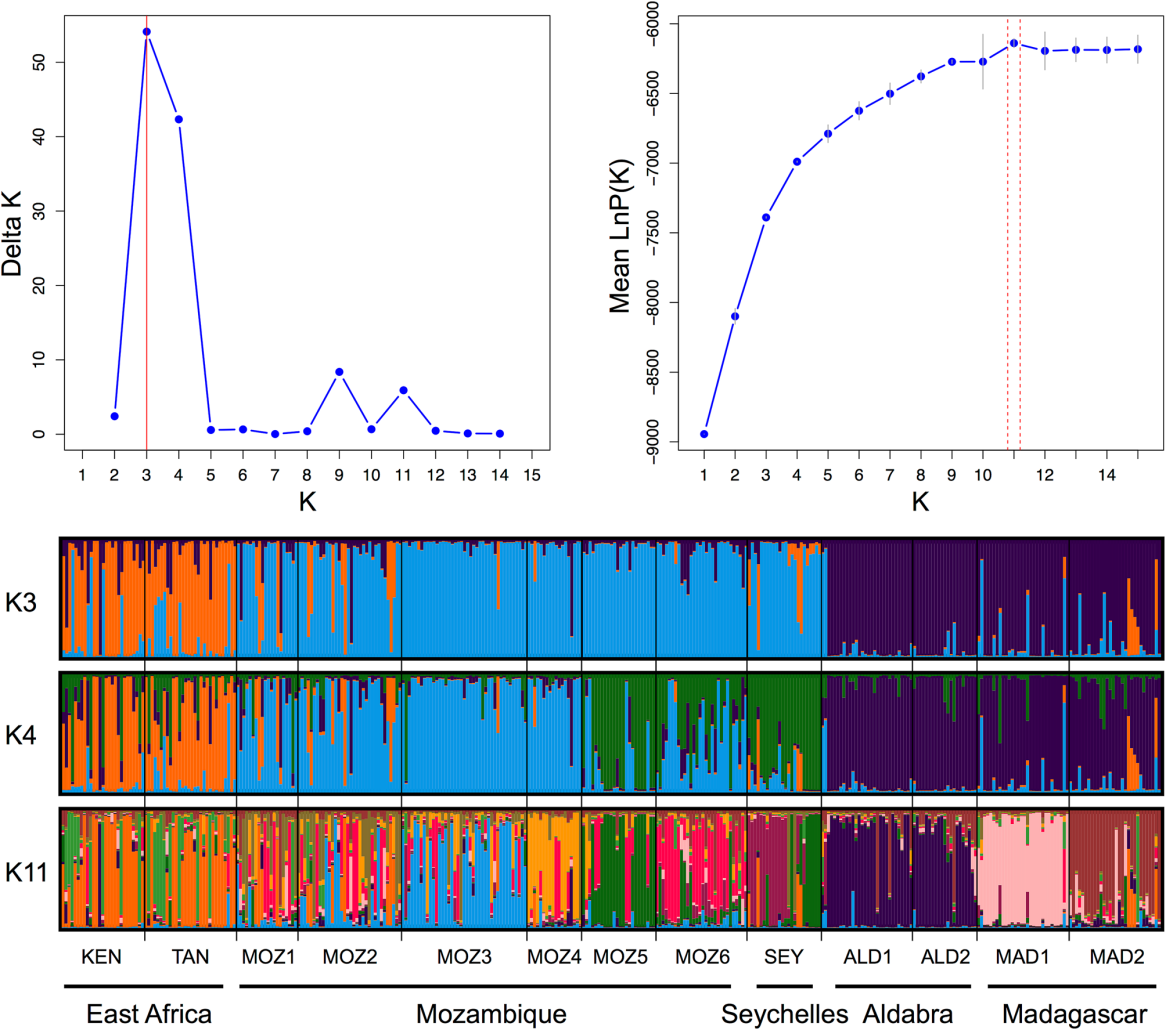
Genetic structure of 13 *Rhizophora mucronata* populations in the Western Indian Ocean region. Each vertical line represents an individual to which genetic clusters are assigned. Structure bar plot considering three (K3) and four (K4) genetic clusters according to the Delta K estimation and considering eleven groups (K11) according to LnPK. These analyses indicate a separate genetic cluster for the remote islands of Aldabra (ALD1, ALD2) and Madagascar (MAD1, MAD2) although the most remote Seychelles population (SEY) appear less differentiated from those on the African coastline. Undoubtedly, a larger allelic diversity of East African populations (KEN and TAN) caused their separated gene pool. *R. mucronata* populations of the Mozambique Channel belong to a well-mixed gene pool. K11 subdivided gene pools of K4, except for East Africa. Population codes are denoted in Table 1.

A Structure analysis according to the Delta K estimation supported K = 3 clusters (Mean LnP(K) = − 7390, Delta K = 54) or nearly as likely also four clusters (Mean LnP(K) = − 6990, Delta K = 42), the latter with more admixed individuals (Fig. 3, K = 3 and K = 4). The same analysis based on the highest LnP(K) value gave eleven groups (Fig. 3, K = 11). These approaches consistently indicated separate genetic clusters for the East African populations (KEN and TAN), and for the remote islands of Aldabra (ALD1, ALD2) and Madagascar (MAD1, MAD2). A larger allelic diversity within East African populations (KEN and TAN) explained their separated gene pool. *Rhizophora mucronata* populations of the Mozambique Channel belong to a well-mixed gene pool, regardless K = 3 or K = 4. The geographically most remote Seychelles population (SEY) appeared less differentiated from those of the African coastline. K = 11 subdivided up to population level, except for East Africa (KEN, TAN), an admixture in Mozambique (MOZ1 to MOZ4) and on Aldabra (ALD1, ALD2). When testing for diversity differences between the K3 clusters, a higher allele diversity (p = 0.011) and gene diversity (p = 0.002) was found in the two East African sites (*A*_r_ = 2.8 and *H*_e_ = 0.410) than in the sites of the Mozambique Channel and SEY (*A*_r_ = 2.3 and *H*_e_ = 0.280) and Aldabra/Madagascar (*A*_r_ = 2.1 and *H*_e_ = 0.200). When considering K4, there were only few individuals (N = 29 out of 359) containing but a limited number of private alleles (PA = 24), all at lowest frequencies such that these private alleles do not explain much of the obtained genetic structure.

Migrate-n was used to estimate the migration pattern along the Mozambique Channel which was best supported by a unidirectional stepping-stone model (Table 4). The directionality, either from North to South or North to South as a whole was not conclusive, due to elevated gene flow in both directions between particular populations in close vicinity, namely MOZ5 to MOZ6 (*N*em = 10.89) and MOZ2 to MOZ1 (*N*em = 11.51). All other *N*em estimates of more distant populations were below 1. Further specific testing on directionality according to the SEC and on a most likely historical source of the Aldabra population and mainland African coast for migration between the Seychelles (SEY); northern Madagascar (MAD2), Aldabra (ALD1) and the African coast (MOZ2) gave the best support for a stepping-stone model (Table 4), with highest, nearly similar, estimated gene flow from the Seychelles to northern Madagascar (*N*em = 0.81) and from Madagascar towards Aldabra (*N*em = 0.78). Historical connectivity between Aldabra and the African coastline reached *N*em = 0.65.

**Table 4:** Comparison of migration models on the directionality along the Mozambique Channel and between the islands Seychelles, Madagascar, Aldabra and East Africa mainland. The model with highest support is highlighted in grey. Connected populations with + means all directions, ←→ refers to bidirectional and → or ← to unidirectionality.

WIO part	Model	Directionality	Connected populations	Bezier log marginal-likelihood	Model choice	Model probability
Mozambique Channel	Panmixia	All	MOZ1 + MOZ2 + MOZ3 + MOZ4 + MOZ5 + MOZ6	− 486,603.29	4	0
	Source-Sink	Bidirectional: North to South and South to North	MOZ1 ←→ MOZ2 ←→ MOZ3 ←→ MOZ4 ←→ MOZ5 ←→ MOZ6	− 452,405.94	3	0
	Stepping-stone	Unidirectional: North to South	MOZ1 → MOZ2 → MOZ3 → MOZ4 → MOZ5 → MOZ6	− 440,940.25	2	0
	Stepping-stone	Unidirectional: South to North	MOZ6 → MOZ5 → MOZ4 → MOZ3 → MOZ2 → MOZ1	− 440,265.68	1	1
Seychelles, Madagascar, Aldabra and African coast	Panmixia	All	SEY + MAD2 + ALD1 + MOZ2	− 885,073,74	2	0
	Source-Sink	Unidirectional: from each island towards mainland	a. SEY → MAD2, SEY → ALD1, SEY → MOZ2 b. MAD2 → ALD1, MAD2 → MOZ2 c. ALD1 → MOZ2	− 886,996,15	3	0
	Stepping-stone	Unidirectional; from island towards mainland	SEY → MAD2 → ALD1 → MOZ2	− 499,291,08	1	1

**Figure 4:**
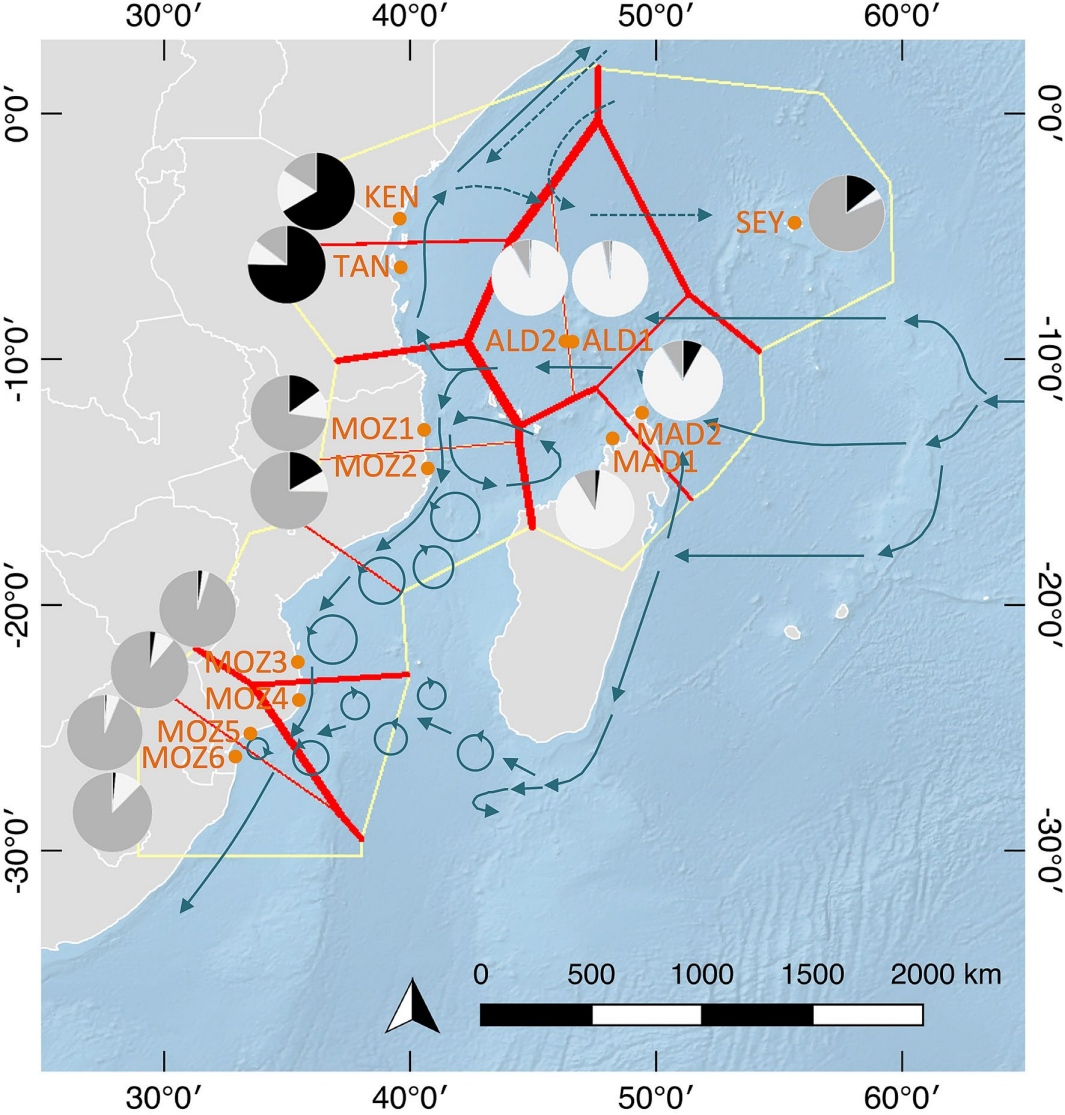
Genetic discontinuities (red lines) as revealed from the Barrier analysis (yellow polygons) and relative proportions of 3 genetic clusters (K = 3) for 13 *Rhizophora mucronata* populations in the Western Indian Ocean. The thickness of the red lines indicates the strength of genetic discontinuity between neighbouring populations. Population codes are denoted in Table 1. The background map was created with the QGIS 3.10.10 software (www.qgis.org), using detailed (1:10 m) ocean bottom relief, land and island polygons, as well as country administrative boundaries provided by Natural Earth (www.naturalearthdata.com).

The most relevant genetic barriers (Fig. 4), between populations along their shared polygon, separated (i) the Aldabra populations from KEN, TAN and MOZ1 on the East African coast (10 first barrier counts out of 18 *F*_ST_ matrices), and to a lesser extent (ii) the Seychelles from Aldabra with seven counts, the Seychelles from East Africa (KEN) with six barrier counts, Aldabra from northern Mozambique (MOZ1) with six barrier counts, and the Southern Mozambican population MOZ4 from MOZ5 with 4 barrier counts. Contemporary kinship values of individual *R. mucronata* trees among populations reached an overall *F*_IJ_ = 0.102, though with large variation between pairs, namely with high values for most pairs of neighbouring populations (*F*_IJ_ ranging from 0.111 to 0.289) except for those pairs where a genetic break was observed (*F*_IJ_ ranging from zero to 0.051). The latter breaks along the East African coastline were between TAN and MOZ1, MOZ4 and MOZ5, MOZ5 and MOZ6. The Seychelles population had zero kinship with Aldabra and Northern Madagascar.

**Figure 5:**
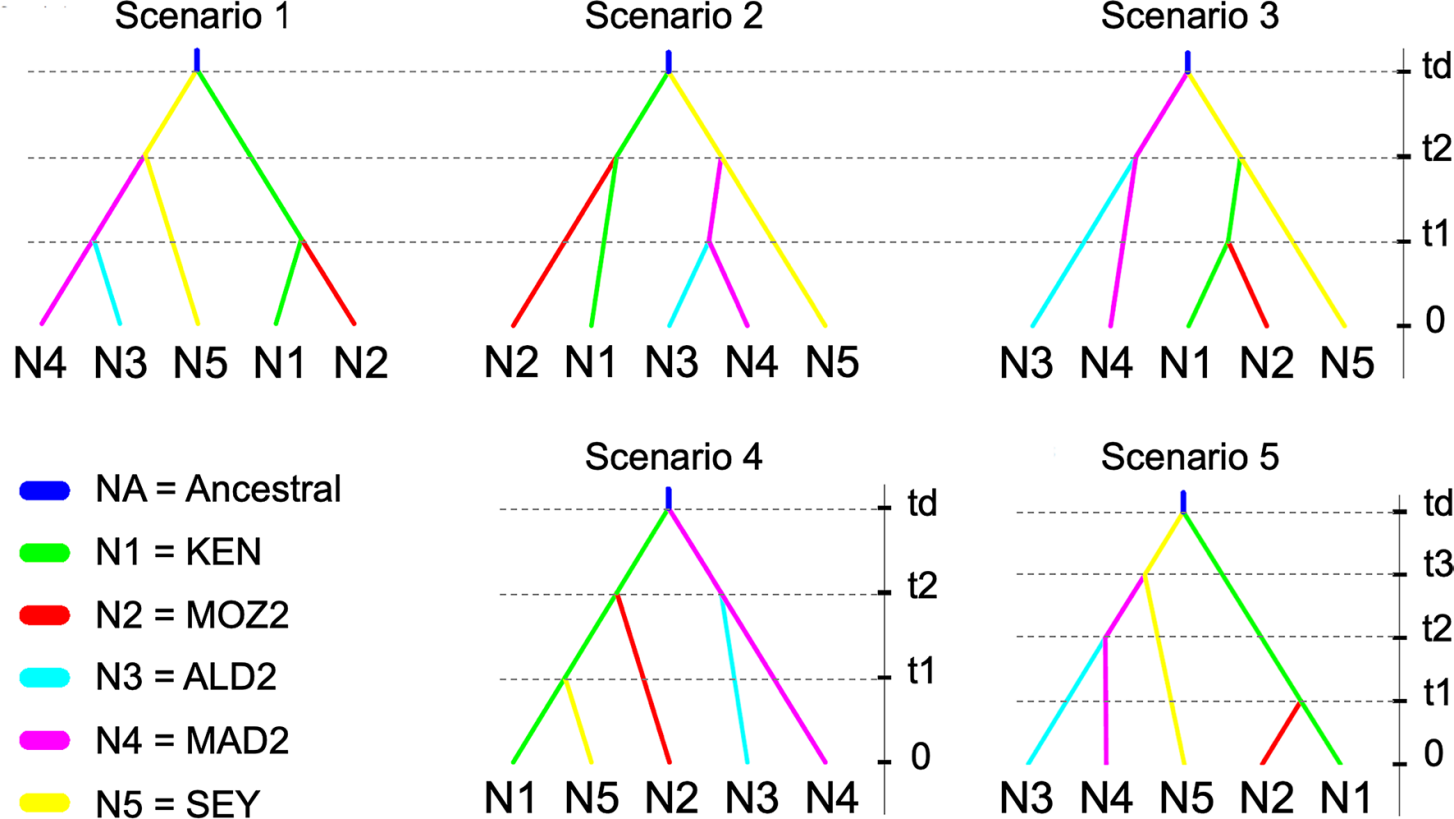
The five scenarios tested for an approximate Bayesian computation (ABC) model implemented in DIYABC ver. 2.0. Five populations with effective population sizes N1 to N5 correspond to KEN, MOZ2, ALD2, MAD2 and SEY, respectively. Scenario 1 represents an early divergence of both the Seychelles and coastal East Africa group with more recent divergence of the Madagascar and Aldabra group; scenario 2 represents an early divergence of both Seychelles and Kenyan populations with more recent divergence of both the Madagascar-Aldabra group and Mozambique Channel Area (MCA) population; scenario 3 represents an early divergence of both the Seychelles and Madagascar populations with a more recent divergence of both the Aldabra population (from Madagascar) and the coastal East Africa group (Kenya and MCA); scenario 4 represents an early divergence of both Madagascan and Kenyan populations with more recent divergence of both the Aldabra and MCA populations, though with the Seychelles as the most recent group originating from East Africa; and scenario 5 includes three period levels with an early divergence of the Seychelles and East Africa group, though with subsequent stepping-stone dispersal from the Seychelles towards Madagascar and then further to Aldabra. NA is the ancestral effective population size and t# represent subsequent time events (not drawn to scale). Scenario 3 considering an early divergence of both Seychelles and Madagascar gave the highest probability.

Comparing the five scenarios of the ABC approach, the highest value of posterior probability (0.338) and 95% confidence interval (CI 0.329–0.346) was obtained for scenario 3 (Fig. 5). This probability value and CI did not overlap with the 95% CI of any other considered scenario (Supplementary Table S2). This indicated a migration history from eastern to western parts of the WIO with a divergence from an ancestral population that was featured by both Seychelles (SEY) and Madagascar (MAD2) populations, though a more recent divergence of the coastal East Africa group (KEN and MOZ2) and Aldabra (ALD2). Absence of significant differences between observed and simulated data in many of the 65 summary metrics (Supplementary Table S3) and the positioning of the observed data within the clustered cloud of simulated data of the PCA (Supplementary Fig. S1) revealed that the selected scenarios fitted the observed data. The median values of the effective population sizes of scenario 3 ranged from 1,500 to 3,080 (Table 5) and median divergence times were estimated as 999 (t1) to 1,980 (t2) generations (Table 5). The ABC simulation indicated that *R. mucronata* populations of East Africa as more recent than those of Madagascar and the Seychelles. The estimated divergence time of *R. mucronata* populations of the eastern WIO island populations (SEY and MAD2) appears approximately twice as old. Considering a generation time of about 20 years for *R. mucronata*, albeit with much potential overlap between generations, the divergence times along the East African coastline could be rather recent and roughly within the Holocene.

**Table 5:** Estimated divergence parameters and their 95% confidence interval based on the logistic estimate of DIYABC for the best supported scenario 3. Scenario 3 represents an early divergence of both the Seychelles and Madagascar populations with a more recent divergence of both the Aldabra population (from Madagascar) and the coastal East Africa group (Kenya and MCA), as shown in Fig. 5. N#: Effective population size; t#: time scale measured in number of generations. Numbers N1-N5 refer respectively to populations KEN, MOZ2, ALD2, MAD2 and SEY, with NA as ancestral population; t1 and t2 refer to increasingly older events, with td as oldest divergence.

Parameter	Mean	Median	Mode	Lower CI	Upper CI
N1	2340.00	2240.00	2020.00	955.00	4130.00
N2	3050.00	3080.00	3170.00	1460.00	4620.00
N3	1680.00	1500.00	1310.00	586.00	3560.00
N4	2010.00	1890.00	1700.00	827.00	3700.00
N5	2210.00	2120.00	2140.00	982.00	3890.00
t1	1090.00	999.00	762.00	321.00	2180.00
t2	2050.00	1980.00	1890.00	953.00	3400.00
td	1910.00	1700.00	1400.00	570.00	4050.00
NA	1800.00	1710.00	935.00	187.00	3720.00

## Discussion

Information retrieved from nuclear microsatellites in *Rhizophora mucronata* from the Western Indian Ocean (WIO) showed a clear genetic structure, largely corresponding to the dominant ocean currents in the region. A major genetic break was apparent between populations on the islands of Aldabra and northern Madagascar, and those on the mainland African coast, whereas the granitic Seychelles (located > 1100 km northeast of Aldabra) showed a slightly separate position. Genetic connectivity usually can be expected up to a few 100 s of km. However, genetic breaks were also found on a smaller spatial scale near the bifurcation of the South Equatorial Current (SEC) and between southernmost populations of the Mozambique Channel. The highest levels of genetic diversity are found along the East African coastline where a bifurcated SEC reaches the continent, whereas the lowest allelic richness was found for populations on remote small islands and in peripheral populations in the southernmost region of the Mozambique Channel. High levels of inbreeding were noticed within most sites, as a result of a mixed mating system of the species.

Prior to our study we considered the null-hypothesis that over a broad geographical scale, *R. mucronata* populations in the WIO would comprise a single evolutionarily significant unit, brought along with the SEC, and originating in a transoceanic manner from either nowadays well-differentiated Southeast Asian^7,21^ or Australian sources^12^. Combined conservative chloroplast DNA and nuclear ITS data indicated a deeper Australian origin^12^, whereas microsatellite markers reflected contemporary genetic structures^7,21^. Within the WIO we found no evolutionary signals over a 3000-km distance, neither in the microsatellite allele sizes (*R*_ST_ = zero) nor in private allele frequencies, which indicates more recent colonization compared to the Eastern Indian Ocean (EIO) and expansion of *R. mucronata* in the region. The share of a large number of common alleles could eventually suggest a stronger influence of environmental conditions during the Last Glacial Maximum (LGM; ca. 22–19 ka BP^39^) on the genetic structure within the WIO. The demographic evolution scenarios that we tested were from diverse locations featured by separate gene pools as shown in our Structure and Barrier analysis. ABC tests indicate that the Seychelles and Madagascar (including Aldabra) gene pool, within the context of the currently studied populations, most likely resulted from ancestral migration events, relatively, about twice as old when compared to the gene pool of more westerly located populations on the East African coastline. All events were estimated at only a few thousand generations ago, hypothesised approximately a timeframe within the Holocene, though the latter needs to be thoroughly tested using large-scale phylogeographic studies and preferably based on maternally inherited chloroplast genome sequences information. We assume that population expansion history of *R. mucronata* in the WIO is characterised by at least two separate ancestral gene pools, namely on the granitic Seychelles and on Madagascar. More recent gene pools may account for the detected patterns along the East African coastline.

Genetic diversity (*A*_R_, *H*_S_, *F*_ST_, *D*_est_) and structure variables (AMOVA testing, Structure, PCoA, Barrier) consistently evidenced a contemporary genetic break between mangrove populations of northern Madagascar and Aldabra versus all other populations, as well as the Seychelles granitic islands, reflecting a complex colonization history of the islands. Coastal areas in northern Madagascar may have been populated by sea-faring propagules transported from the east, via the SEC. Despite the westward directionality of the SEC, broadly along these Madagascan populations, this would require dispersal over 6000 + kilometres, as the most proximate large mangrove populations are situated in Southeast Asia or northern Australia. However, direct and reciprocal dispersal across the Indian Ocean may occur via the SEC and seasonally reversing monsoon currents, particularly for propagules with longer floating and viability periods^40^, such as found in *R. mucronata*^9^. The latter hypothesis of reciprocal dispersal should be tested with materials from the northern part of the WIO. When approaching the central eastern coast of Madagascar, the SEC branches into the North-East Madagascar Current (NEMC) and the South-East Madagascar Current (SEMC) (^41^; Fig. 1). The NEMC flows northward and continues along the northern tip of Madagascar towards the African mainland where it bifurcates into a southward flow and the East Africa Coastal Current (EACC), flowing northward (Fig. 1).

The characteristics of these currents, the genetic similarities and migration scenario’s between *R. mucronata* populations in Aldabra and northern Madagascar strongly suggest that the Aldabra Atoll was populated from nearby sources on northern Madagascar. Aldabra populations are extremely isolated by their position on a small and remote island, far away from more extensive and continuous mangrove stands. Moreover, mangroves occur within the lagoon where conditions are more favourable for establishment^34^. The unfavourable conditions of the atoll’s outer rim could drastically reduce the establishment of propagules from source locations such as northern Madagascar, located about 420 km southeast of the Aldabra Atoll. Hence, any input of genetic material from other populations via long-distance dispersal is assumed to be seldom throughout its recent history. Allelic data shows that colonisation occurred on more than one occasion, as suggested also for terrestrial vertebrates such as giant tortoises^42^. These cycles of recolonization were interrupted by variations in sea level, which have strongly affected the atoll’s land area and environment, with a complete submergence during the last interglacial period (ca. 125 ka BP)^43^.

At least more than a single propagule reached the granitic Seychelles or, alternatively, different cycles of colonisation may have occurred, because there, *R. mucronata* populations contain more than two alleles for most polymorphic loci, thus beyond the maximum allele diversity that a single diploid founder may bring along. Allele size differences within the Seychelles were not larger than for other WIO populations, indicating either an admixture of populations or in-situ mutations to explain their multiple allele status. While the SEC provides an important vector for the transoceanic transport of propagules, previous studies reported that the zonal flow of the SEC creates a barrier for connectivity between the granitic Seychelles and more southerly positioned populations^44^. This is consistent with a genetic “isolation” of the granitic Seychelles from Aldabra and Northern Madagascar, although various migration models indicated a historical role of the Seychelles in the connectivity between islands. Moreover, the ABC analysis supported both the Seychelles and Madagascar as an ancestral divergence, which might explain the support of a unidirectional model in Migrate-n albeit with very low gene flow values. Mangrove populations on the Seychelles are likely maintained by propagules from sources of the northern part of the WIO, via the SECC. During the winter monsoon, the Somali Current flows southward, meets the northward flowing EACC, supplying water for the eastward flowing SECC^41^. This confluence and eastward flow occur at latitudes of the granitic Seychelles and enables propagule transport from mainland mangrove populations to these islands. This could not be detected from nuclear microsatellite markers and additional chloroplast sequence information could provide valuable information for unravelling patterns of connectivity between the Seychelles, Madagascar and other African populations. Nuclear genes and chloroplast sequences of *Bruguiera gymnorrhiza* (L.) Lamk. indicated a relatedness of Madagascar populations to those of the EIO^45^ and cpDNA of the widespread *Xylocarpus granatum* J. Koenig showed a most common South China Sea haplotype in several WIO populations^46^.

A north-to-south gradient of allelic richness characterised the African mainland sites with highest diversity values in Kenya and Tanzania, north of the SEC bifurcation. The mangrove sites influenced by the EACC appear to have received and sustained more migrants than other areas although, based on our ABC analysis, this appeared to have occurred during more recent rather than ancestral times. The SEC very likely sustained these populations with a historically accumulated input of migrants from remote easterly mangrove populations, located within or beyond the WIO. While this scenario could suggest the expectation to observe the highest genetic diversity in (northern) East Madagascar, spatial differences in the availability of suitable geomorphic and sedimentary settings for mangrove establishment may present higher chances of opportunistic colonization on the East African coast compared to the higher-energy coast of East Madagascar. Importantly, migrants from eastern populations may have also arrived via populations in the Arabian Sea and Gulf of Bengal, as suggested in previous studies^33,40^. However, the spatial density and spread of our sample sites did not allow us to test the latter scenario.

Over a 225 km distance, KEN and TAN were the most connected *R. mucronata* populations with the highest kinship values, and even appeared as a single gene pool from the Structure outcome at K11. Similar evidence of high connectivity alongside this part of the East African coast was also observed in other taxa, such as the stony coral *Acropora tenuis*^47^ and the seagrass *Thalassia hemprichii*^48^. Likewise, a higher genetic diversity and stronger connectivity was observed for the populations of northern Mozambique (MOZ1 and MOZ2) as compared to the more distant southernmost populations in the Mozambique Channel. Overall, the core region of mangrove populations, situated at latitudes of the SEC (KEN, TAN, MOZ1, MOZ2), seems to harbour the most diverse *R. mucronata* populations. Such enhanced diversity levels in a zone of largest and most direct—perpendicular—SEC influence could become a more general hypothesis because *Avicennia marina* mangroves also showed highest allele diversity in Kenya and Tanzania in comparison to South Africa^32^. Strong genetic differentiation between populations along the northern East African coast (TAN and KEN) and populations located in the Mozambique Channel (MOZ1, MOZ2) corresponds with the SEC bifurcation. These distinct gene pools most evidently originate from a historically different accumulation of migrants and the opposite directionality of ocean currents hampering mutual propagule exchange^7,16,25,30,49,50^. One may assume that besides the SEC sorting migrants at bifurcation, subsequent differentiation and sorting in a stepping-stone manner may have occurred northwards and southwards along the East African coastline.

The geographical position of MOZ2 at the southern bifurcation of the SEC and starting point of eddies in the Mozambique Channel could be hypothesised as a crossroad. A genetic connectivity from the eastern part of the Mozambique Channel (northern Madagascar or Aldabra) towards MOZ1 and MOZ2 could not be detected with certainty, but only indirectly inferred; the Migrate-n analysis indicated MOZ2 as a population with detectable *N*_e_m input. Experimental and modelling studies in the Mozambique Channel have demonstrated the capability of cross-channel transport between Mozambique and Madagascar within 19–30 days, likely facilitated by the frontal zones between eddies and the associated interstitial waters of the turbulence field^49,50^. This potential for cross-channel transport was also demonstrated in a large-scale WIO population genetic study of the seagrass *T. hemprichii*, suggesting Madagascar as a potential source for main African populations^48^. The remote Seychelles population could be occasionally connected to northern Madagascar and to the coast of the African mainland, though the input source of migrants will be far too limited to leave a detectable trace in a vast area of existing mangrove forests. Whereas it appears that the Seychelles would share a common gene pool with MOZ1 and MOZ2 for K3, all SEY individuals were 100% assigned to their own population.

Within the Mozambique Channel, *R. mucronata* populations are poorly differentiated and all share a common genetic cluster at K3. This gradient and connectivity is likely to be maintained by the generally southward flowing current along the Mozambique coast^51^ and the anti-cyclonic circulation pattern in the region, which is believed to facilitate an almost random dispersal and homogenise populations along the East African coast^53,54^. Despite the dominance of southward flow in the Mozambique Channel, propagules might be transported both southward and northward through the Channel within periods of 51–207 days, via the frontal flow field between eddies^51^. These time spans are within the range of buoyancy and viability periods observed in *R. mucronata* propagules^9^. Along the southward component of the SEC, and further south along the Mozambique Channel, a gradient was apparent in *R. mucronata* populations for their lowered allelic richness and gene diversities, increased inbreeding levels, number of fixed loci and an admixed assignment of individuals for genetic clustering (cf. K4 and K11). The connectivity between neighbouring populations decreased (cf. higher *F*_ST_, D_est_) and kinship values dropped to zero between MOZ4 and MOZ5, also indicated by low gene flow estimates and a genetic break situated on the Delagoa Bight. An extensive chain of mangrove forest can be found from approximately the northern Kenyan border further southwards up to about 50 km south from the mouth of River Save on the Sofala bank in Mozambique^34^. However, beyond this point, mangrove forests line the coasts in a less continuous manner and are mostly found in more sheltered inlets^34^. The populations MOZ3 to MOZ6 are situated in this area of non-contiguous mangrove forests. MOZ3 lies only 100 km south of a 1900 km^2^ mangrove stand fringing the Sofala Bank, which is considered the largest mangrove area in the region^55^ and hence might provide a higher number of propagules for dispersal. MOZ4, MOZ5 and MOZ6 are located ca. 300–800 km from the extensive Sofala Bank mangrove stand. Therefore, we hypothesise that migrants from the Sofala Bank favour higher diversity in MOZ3 and MOZ4 when compared to the Delagoa Bight populations, MOZ5 and MOZ6.

The genetic break between MOZ4 and MOZ5, despite their geographic proximity, can be explained by the dynamic hydrography near the Delagoa Bight area. MOZ3 and MOZ4 are located on a protruding land area, where the continental shelf is practically inexistent. A jet current (i.e., fast flowing narrow current) passes just along MOZ4^51^, flowing southward without entering the Delagoa Bight. Additionally, the Delagoa Bight is characterised by mesoscale features such as countercurrents and eddies^56^ and consists of a large extended shelf area where lies a quasi-permanent lee eddy^57^. These features may retain propagules from populations in this area (MOZ5 and MOZ6) and prevent dispersal offshore^58^. A sharp gradient in genetic diversity was also observed in the spiny lobster *Panulirus homarus* and separated even evolutionary units of subspecies at the Delagoa Bight^59^. It is most probable that both the lee eddy of the Delagoa Bight and the jet current prevent propagules from northward populations to strand in the Delagoa Bight. Subsequently, the mixed mating system of *R. mucronata* may promote high inbreeding values and many fixed loci as were noticed in the well-connected populations MOZ5 and MOZ6.

Inbreeding within nearly each *R. mucronata* site is most likely the result of geitonogamy as in *R. racemosa*^60^. This can blur the determination of a genetic structure at the population level because occasional fixation of private alleles might lead to enforced clusters of gene pools. However, this situation was not observed in the WIO because *R. mucronata* populations shared common alleles throughout the region, such that allelic richness (*A*_R_) and gene diversities (*H*_E_ and *D*_est_) determined the detectable patterns of connectivity at population level. High within-site kinship values and estimated selfing rates can be explained from their high frequencies of most common alleles. *Rhizophora mucronata* is a self-compatible species^61^. Similar inbreeding levels for *R. mucronata* have been reported in Southeast Asia and were attributed to the reproductive biology and dispersal strategies of the species^16^. Interestingly, lower inbreeding values were found in populations of *R. racemosa in* West Africa^15^ than in *R. mucronata*. *Rhizophora racemosa*’s fruits resemble those of *R. mucronata*, which suggests that besides reproductive strategy, propagule morphological traits, and dispersal strategies, other factors such as local topography and species zonation (landward/seaward) may influence the local inbreeding level of these coastal populations^13^.

In conclusion, our study highlighted that *R. mucronata* of the WIO should be considered as a recent colonization (in comparison to the EIO) with an expansion zone that has particularly led to areas of unicity, of higher diversity, of inbreeding, and of contrasting connectivity even over short distances, which all are relevant in a transboundary context. Anticipating the ever-increasing need for international cooperation regarding the conservation and management of dispersive coastal and marine species, we encourage the integration of available genetic data and models to further increase our understanding of the interplay between the ecology and evolution of mangroves, large-scale (transnational) connectivity, and aspects such as species distributions and long-term persistence.

## Material and methods

### Plant materials and study area

A total of 359 *Rhizophora mucronata* samples were collected in 13 locations (Fig. 1, Table 1) of which 8 populations were located along a 3465 km stretch of the East African continent and 5 populations on islands in the WIO. For each sample site, fresh leaves were collected along a linear transect of 18–41 individual adult trees and stored in individual bags with silica gel for transport. Transect length depended on the size of the local area of mangrove forest for sampling trees that were positioned as far away from each other as possible in order to increase the probability to sample the total population genetic diversity. Distance between individual trees ranged from 30 to 100 m.

### Microsatellite analysis

Total genomic DNA extraction was performed on 30 mg of dried leaf material using the E.Z.N.A. SP Plant DNA kit (Omega bio-tek, Norcross, GA, USA) for subsequent microsatellite analysis. A total of 50 previously published microsatellite primer pairs of *Rhizophora stylosa*, *R. racemosa* and *R. mucronata* were tested for polymorphism of *R. mucronata* samples^31,62–67^. Seventeen primers were polymorphic and amplified in two multiplex PCRs. Multiplex 1 consisted of Rrace12, Rrace14, Rrace15, Rrace17, Rrace18^31^, RM11^63^ and RmBra27^67^, multiplex 2 contained RM102, RM112, RM114, RM116^65^, RMu21, RMu54^66^, Rhst01, Rhst11, Rhst13^62^ and RS78^64^. Multiplex Polymerase Chain Reactions (PCR) were performed with the QIAGEN Multiplex PCR kit and consisted of 12.5 μL reactions with 2.5 μL H_2_O, 6.25 μL of Qiagen Multiplex PCR Master Mix, 1.25 μL primer mix and 2.5 μL DNA. The primer mix consisted of 2 μM of each fluorescence-labelled (VIC, PET, 6-FAM or NED) microsatellite primer (Life Technologies, Foster City, CA, USA). PCRs were carried out in a BIO-RAD T100 thermal cycler (Bio-Rad laboratories, Hercules, CA, USA), starting with an initial activation step of 15 min at 95 °C, followed by 35 cycles of 30 s at 94 °C, 90 s at 57 °C and 90 s at 72 °C, and ended with a final extension of 10 min at 68 °C. Fragment analysis was done by Macrogen Inc., after which allele sizes were assessed with the software Genemarker 2.4.0 (Softgenetics, State College, PA, USA).

### Quality of microsatellite data

Prior to full data analysis, all pairs of microsatellite loci were tested for linkage disequilibrium with FSTAT 2.9.3 software^68^ and no significant genetic linkage (p < 0.05) was found. No scoring errors or large allele size dropouts were indicated using Micro-Checker^69^, whereas a general excess of homozygotes for many loci suggests the presence of null alleles. This test, however, does not consider the effect of inbreeding situations and therefore INEST 2.2^70^ was applied as a Bayesian approach for estimating both null alleles and inbreeding simultaneously^71^. The model was run with 50,000 burn-ins and 500,000 cycles and supported for each population a model including inbreeding rather than a null allele model. Three parameters were used for the model comparison: n, null alleles; f, inbreeding; b, genotyping failure. With these three parameters, six models are composed: n, b, nf, nb, bf, nfb (e.g., nf means the model includes null alleles and inbreeding but does not include genotyping failures). The six models were run with 50,000 burn-ins and 500,000 cycles for each population. A comparison of these models with the lowest Deviance Information Criterion (DIC) outperforming the other models, selected nf (in 8 populations), nbf (in 4 populations) and bf (in 1 population) as best fit, hence putting inbreeding instead of null alleles as a most likely explanation for the lack of heterozygotes. The probability of identity, using Genalex 6.5^72^, was checked for a combination of all loci and ranged from 5 × 10^–3^ to 9 × 10^–8^. Multilocus repeats were seldom, namely 16 repeats for 9 MLG’s out of 359 samples. All repeated MLG’s were kept for further analysis, considering the INEST indications of inbred situations.

### Allele and gene diversity

The percentage of polymorphic loci per population, mean number of alleles (*A*_M_), effective number of alleles (*A*_E_), observed heterozygosity (*H*_O_) and unbiased expected heterozygosity (*H*_E_) were calculated using Genalex. Total number of alleles (*A*), allelic richness (*A*_R_), deviation from the Hardy–Weinberg Equilibrium and the within population inbreeding coefficient (*F*_IS_) per population were obtained with FSTAT. Deficiency of heterozygotes was assessed after Bonferroni correction using *F*_IS_ values with 221,000 randomisations. The selfing rate was estimated and based on a standardised identity disequilibrium, assuming a mixed mating model using spagedi 1.5^73^. A population assignment (with leave-one-out option) was done for their proportion of 'self' or 'other' populations with Genalex. The overall pairwise kinship coefficient (*F*_IJ_)^74^ was estimated for all comparisons at the ‘within population level’ using spagedi. To strengthen the evidence at both large and small geographical scales, we intentionally conducted most approaches at the individual level, because population level analysis might become skewed in case of strong inbreeding.

### Genetic structure

An analysis of Molecular Variance (AMOVA-*F*_ST_) was performed with Genalex. Additionally, all combinations within a hierarchy of 2, 3 and 4 groups were tested for highest % variance among regions (*F*_RT_). To estimate the effects of microsatellite allele sizes, an AMOVA-*R*_ST_ was compared to AMOVA-*F*_ST_. Pairwise genetic differentiation (*F*_ST_) and allelic differentiation (*D*est) between all populations were estimated and tested for significant differences (999 permutations) using Genalex. A principal coordinate analysis (PCoA) at individual level based on genotypic distances was performed in Genalex. The underlying pattern of genetic structure was determined with Structure 2.3.2^75^, using the admixture model with correlated allele frequencies without prior population information. We carried out 10 replications for 1 ≤ K ≤ 15 with 1 × 10^5^ Markov Chain Monte Carlo (MCMC) iterations and 1 × 10^4^ burn-in. The optimal K was inferred with the ΔK statistic^76^ and LnPK using Structure Harvester^77^ calculated with Structureselector^78^. Two-sided tests for comparisons among three groups (i.e., the obtained K = 3 clusters from Structure) were performed (1000 permutations in FSTAT) to test for differences in their allelic richness, heterozygosity, inbreeding and differentiation. Private alleles were checked for the K = 3 and K = 4 clusters in Genalex.

To detect whether there is a decrease in connectivity among populations with increasing geographic distance, a Mantel test was performed (9,999 permutations) in Genalex 6.5 using Euclidean distances between sites. Geographic distances were log transformed while *F*_ST_/(1 − *F*_ST_) was used for the genetic distance matrix. To test for genetic IBD at the individual level, an analysis of the pairwise kinship coefficients (*F*_IJ_)^74^ was done using spagedi, namely a spatial autocorrelation for different classes of geographic distances (260, 500, 1000, 2000 km, 1-sided test, 1,000 randomizations) with estimation of an ln-transformed b-slope over the full range of 3008 km. The overall pairwise kinship coefficient was estimated for all comparisons at the ‘among population level’. The software Barrier 2.2^79^ was used to detect the location of sharp genetic changes between neighbouring populations on basis of one overall pairwise *F*_ST_ matrix and 17 pairwise *F*_ST_ matrices of every microsatellite locus allowing a maximum of two barriers per matrix. To test for these potential genetic breaks specifically between neighbouring populations, an analysis of the pairwise kinship coefficients (*F*_IJ_)^74^, was done for each case of only among population’ pairwise estimation (1-sided test, 1,000 randomizations, whole population kept as reference) using spagedi.

Migrate-n^80,81^ was used to estimate the mutation-scaled population sizes (Theta) and immigration rates (M). The Brownian model was tested locus by locus along with the product of distributions of all loci for all individuals in a population. Uniform prior distribution settings (min, max, delta) were Theta = 0.0, 10.0, 0.1 and for M = 0.0, 100, 10.0. The number of recorded steps was 10^6^ at a sampling frequency of 10^3^ after an initial burn-in, computing two replicate chains, and using the Bezier thermodynamic integration^81^ for calculation of the Bayes factors from marginal likelihoods giving model probabilities. The effective number of immigrants per generation (*N*_e_m) was calculated as [Theta × M]/4^28^. Panmixia, bidirectional and unidirectional stepping-stone historical migration/expansion models were used for testing the hypothesis of a North to South migration in the Mozambique Channel (MOZ1 to MOZ6). Panmixia, source-sink or stepping-stone models were considered to test a specific hypothesis on directionality for migration among the island populations Seychelles (SEY), northern Madagascar (MAD2) and Aldabra (ALD1), and towards the African mainland (MOZ2).

The demographic history of divergence between *R. mucronata* populations of the WIO was carried out on seventeen microsatellites considered as a single group with dinucleotide repeats, using the Approximate Bayesian Computation (ABC) approach implemented in DIYABC ver. 2.0^82^. The aim of this analysis was to answer a main phylogeographic question that was raised from the genetic Structure and the Migrate-n outcome, namely about the ancestral origin of eastern WIO island populations (granitic Seychelles, Madagascar and Aldabra) and of coastal African populations (East to Southern Africa). Therefore, we considered representative allelic rich *R. mucronata* populations within each of the of five regions, namely KEN, MOZ2, ALD2, MAD2 and SEY to conduct a model where we specifically conceptualised five scenarios of demographic history (Fig. 5). The first three scenarios allowed inferring whether the early divergences within the WIO were either from the Seychelles, Madagascar or the coastline of the African mainland. Scenario 1 represents an early divergence of both the Seychelles and coastal East Africa group with more recent divergence of the Madagascar and Aldabra group; scenario 2 represents an early divergence of both the Seychelles and Kenyan populations with more recent divergence of both the Madagascar-Aldabra group and Mozambique Channel Area (MCA); scenario 3 represents an early divergence of both the Seychelles and Madagascar populations with a more recent divergence of both the Aldabra population (from Madagascar) and the coastal East Africa group (Kenya and MCA). An additional fourth scenario was considered to evaluate whether populations from the Seychelles could have diverged from the main continent more recently and subsequently brought along with the SECC; scenario 4 represents an early divergence of both Madagascar and Kenyan populations with more recent divergence of both Aldabra and MCA populations, though with the Seychelles as the most recent group originating from East Africa; finally, scenario 5 highlighted a potential divergence from the Seychelles, over Madagascar towards Aldabra as was obtained from Migrate-n. Scenario 5 includes three period levels with an early divergence of the Seychelles and coastal East Africa group, though with subsequent stepping-stone dispersal from the Seychelles towards Madagascar and then further towards Aldabra. In all models we constantly kept Aldabra as being split from Madagascar.

In all scenarios, t# represents the time scale measured in number of generations (largest numbers refer to oldest events) and N# represents the effective size of the corresponding populations during the indicated time period. We used default prior values for all parameters, except for the maximum population size and maximum values of time scale (5,000 instead of 10,000 default values) based on the outcome of the preliminary test runs of prior distributions. Summary statistics of 65 variables (mean number of alleles, mean gene diversity, mean allele size variance, *F*_ST_, and (dµ)^2^ distance) were considered for each population or pairwise comparison of samples. Five million simulation data sets were run for the prior distribution and the most-likely scenario was obtained from a comparative assessment of their posterior probabilities. One million simulated data sets were used to compute posterior, of which 10,000 were used in the local regression. From the posterior 1,000 data sets were simulated. The goodness-of-fit was checked through principal component analysis (PCA) using the ‘model checking’ option. The posterior distribution of parameters (N# and t#) was estimated.

## Supplementary information

Supplementary information.
