# Supplementary material for: Expansion of the mangrove species Rhizophora mucronata in the Western Indian Ocean launched contrasting genetic patterns
Source: Sci Rep. 2021 Mar 2;11:4987. doi: 10.1038/s41598-021-84304-8 (PMC7925526; doi:10.1038/s41598-021-84304-8)

Supplementary information for:

Expansion of the mangrove species *Rhizophora mucronata* in the Western Indian Ocean launched contrasting genetic patterns

Ludwig Triest[1,‡,*], Tom Van der Stocken[1,‡], Dennis De Ryck[1], Marc Kochzius[2], Sophie Lorent[1], Magdalene Ngeve[1,3], Hajaniaina Andrianavalonarivo Ratsimbazafy[1,2,4], Tim Sierens[1], Rosa van der Ven[2,5], Nico Koedam[1]

1. Ecology and Biodiversity, Vrije Universiteit Brussel, Pleinlaan 2, 1050 Elsene, Brussels, Belgium
2. Marine Biology, Vrije Universiteit Brussel, Pleinlaan 2, 1050 Elsene, Brussels, Belgium
3. Department of Plant Sciences and Landscape Architecture, University of Maryland, College Park, MD 20742, United States
4. Laboratory of Systems Ecology and Resource Management, Département de Biologie des Organismes, Université Libre de Bruxelles - ULB, Av. F.D. Roosevelt 50, CPi 264/1, 1050, Brussels, Belgium
5. Marine Animal Ecology Group, Wageningen University, Wageningen, The Netherlands

[‡]These authors contributed equally to this work
*Corresponding author: ltriest@vub.be

Table S1. Pairwise F_{ST} estimates of 13 *Rhizophora mucronata* populations of the Western Indian Ocean. Neighbouring sites appear fully connected as can be expected (MOZ1 vs MOZ2; and ALD1 vs ALD2). Strongest differentiation was obtained for Aldabra (ALD1, ALD2) or Madagascar (MAD1, MAD2) against all other sites. Pairwise F_{ST} values are given below and *D*est values above the diagonal. All values are significant at $p < 0.001$, unless indicated otherwise: ** significant at $p < 0.01$, and ns: not significant.

	KEN	TAN	MOZ1	MOZ2	MOZ3	MOZ4	MOZ5	MOZ6	SEY	ALD1	ALD2	MAD1	MAD2
**KEN**	-	0.075	0.114	0.139	0.289	0.254	0.247	0.213	0.204	0.195	0.190	0.197	0.149
**TAN**	0.091	-	0.176	0.203	0.291	0.266	0.214	0.205	0.214	0.259	0.240	0.268	0.204
**MOZ1**	0.139	0.210	-	0.001ns	0.069	0.076	0.152	0.073	0.149	0.149	0.143	0.117	0.106
**MOZ2**	0.188	0.260	0.002ns	-	0.052	0.078	0.166	0.082	0.168	0.162	0.159	0.118	0.124
**MOZ3**	0.354	0.365	0.134	0.109	-	0.072	0.156	0.069	0.156	0.205	0.209	0.179	0.188
**MOZ4**	0.304	0.329	0.133	0.151	0.159	-	0.138	0.083	0.176	0.176	0.197	0.203	0.174
**MOZ5**	0.365	0.343	0.314	0.333	0.345	0.354	-	0.043	0.103	0.178	0.150	0.235	0.136
**MOZ6**	0.298	0.299	0.150	0.171	0.162	0.200	0.147	-	0.091	0.144	0.115	0.161	0.092
**SEY**	0.248	0.267	0.218	0.256	0.268	0.290	0.248	0.189	-	0.198	0.203	0.238	0.197
**ALD1**	0.275	0.346	0.262	0.287	0.367	0.344	0.417	0.313	0.336	-	0.015**	0.096	0.047
**ALD2**	0.284	0.344	0.274	0.301	0.391	0.404	0.426	0.292	0.364	0.051	-	0.098	0.028
**MAD1**	0.307	0.384	0.252	0.249	0.360	0.415	0.529	0.369	0.409	0.258	0.294	-	0.073
**MAD2**	0.238	0.309	0.217	0.246	0.358	0.357	0.372	0.238	0.347	0.135	0.099	0.220	-

Table S2. Posterior probability of each of the five divergence scenarios and their 95% confidence interval based on the logistic estimate of DIYABC. The model of five populations with five scenarios are drawn in Figure 5.

Scenario	Posterior probability	95% CI [lower-upper]
1	0.140	[0.135 - 0.145]
2	0.145	[0.139 - 0.150]
3	0.338	[0.329 - 0.346]
4	0.275	[0.267 - 0.283]
5	0.103	[0.098 - 0.108]

Table S3. Numerical results of model checking (scenario 3) in terms of one sample and two sample summary statistics (65 in total) of microsatellite loci. NAL: mean number of alleles across loci; HET: mean gene diversity across loci; VAR: mean allele size variance across loci; N2P: mean number of alleles across loci (for two samples); H2P: mean gene diversity across loci (for two samples); V2P: mean allele size variance across loci (for two samples); FST: Differentiation between two samples; DM2: $(d\mu)^2$ distance for microsatellites between two samples. * indicates proportions of simulated data with value lower than 5% or greater than 95% of the observed data set; ** for 1% and 99%, respectively.

Summary statistics	Observed value	Proportion (simulated<observed)
NAL_1_1	2.7059	0.2560
NAL_1_2	2.8824	0.1405
NAL_1_3	2.4118	0.2630
NAL_1_4	2.2941	0.0750
NAL_1_5	2.1765	0.0300*
HET_1_1	0.4161	0.6095
HET_1_2	0.3244	0.1695
HET_1_3	0.3260	0.4245
HET_1_4	0.2279	0.0505
HET_1_5	0.2010	0.0215*
VAR_1_1	1.4376	0.8875
VAR_1_2	1.1713	0.7950
VAR_1_3	0.8893	0.7565
VAR_1_4	1.3696	0.8840
VAR_1_5	0.5419	0.3305
N2P_1_1&2	3.3529	0.1055
N2P_1_1&3	3.2353	0.1185
N2P_1_1&4	3.2353	0.0845
N2P_1_1&5	2.9412	0.0195*
N2P_1_2&3	3.3529	0.0845
N2P_1_2&4	3.2941	0.0395*
N2P_1_2&5	3.1176	0.0190*
N2P_1_3&4	3.1176	0.0905
N2P_1_3&5	2.9412	0.0325*
N2P_1_4&5	2.8824	0.0170*
H2P_1_1&2	0.4033	0.3465
H2P_1_1&3	0.4337	0.3900
H2P_1_1&4	0.3742	0.1830
H2P_1_1&5	0.3433	0.1245
H2P_1_2&3	0.3816	0.1935
H2P_1_2&4	0.3371	0.0950
H2P_1_2&5	0.3115	0.0665
H2P_1_3&4	0.3419	0.1540
H2P_1_3&5	0.3282	0.0860
H2P_1_4&5	0.2339	0.0100**
V2P_1_1&2	1.5228	0.8840
V2P_1_1&3	1.2159	0.7210
V2P_1_1&4	1.7733	0.8860

V2P_1_1&5	1.4507	0.8330
V2P_1_2&3	1.3108	0.7440
V2P_1_2&4	1.5719	0.8430
V2P_1_2&5	1.1521	0.6880
V2P_1_3&4	1.4468	0.8500
V2P_1_3&5	1.1098	0.6525
V2P_1_4&5	1.1831	0.6870
FST_1_1&2	0.1870	0.7895
FST_1_1&3	0.2463	0.3040
FST_1_1&4	0.3037	0.6375
FST_1_1&5	0.2627	0.6645
FST_1_2&3	0.2553	0.3870
FST_1_2&4	0.2930	0.6545
FST_1_2&5	0.2551	0.7195
FST_1_3&4	0.3428	0.7520
FST_1_3&5	0.3626	0.7425
FST_1_4&5	0.1474	0.0435*
DM2_1_1&2	1.0568	0.9500*
DM2_1_1&3	0.2870	0.1345
DM2_1_1&4	1.5823	0.8370
DM2_1_1&5	2.2081	0.9510*
DM2_1_2&3	1.0858	0.6590
DM2_1_2&4	1.2529	0.7525
DM2_1_2&5	1.1600	0.8480
DM2_1_3&4	1.1919	0.7875
DM2_1_3&5	1.7173	0.8250
DM2_1_4&5	0.9347	0.6030

Fig. S1. Principal Component Analysis (PCA) of the model checking for the selected scenario 3 of the demographic history model as estimated in DIYABC.

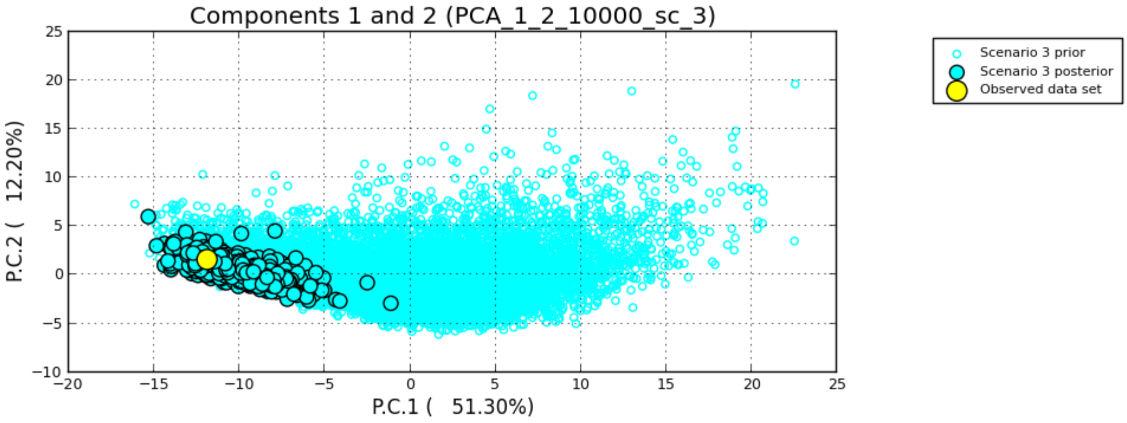

Supplement: Supplementary file 1 — Supplementary information. [file 41598_2021_84304_MOESM1_ESM.pdf]
